# Prion Diagnosis: Application of Real-Time Quaking-Induced Conversion

**DOI:** 10.1155/2017/5413936

**Published:** 2017-05-17

**Authors:** Hae-Eun Kang, Youngwon Mo, Raihah Abd Rahim, Hye-Mi Lee, Chongsuk Ryou

**Affiliations:** ^1^Division of Foreign Animal Disease, Animal and Plant Quarantine Agency, Gimcheon, Gyeongsangbuk-do 39660, Republic of Korea; ^2^Department of Pharmacy and Institute of Pharmaceutical Science and Technology, Hanyang University, Ansan, Gyeonggi-do 15588, Republic of Korea; ^3^UCL School of Pharmacy, University College London, London WC1N1AX, UK

## Abstract

Prions composed of pathogenic scrapie prion protein (PrP^Sc^) are infectious pathogens that cause progressive neurological conditions known as prion diseases or transmissible spongiform encephalopathies. Although these diseases pose considerable risk to public health, procedures for early diagnosis have not been established. One of the most recent attempts at sensitive and specific detection of prions is the real-time quaking-induced conversion (RT-QuIC) method, which measures the activity of PrP^Sc^ aggregates or amyloid formation triggered by PrP^Sc^ seeds in the presence of recombinant PrP. In this review, we summarize prions, prion diseases, and current approaches to diagnosis, including the principle, conditions for assay performance, and current diagnostic applications of RT-QuIC.

## 1. Prions

Prions, defined as proteinaceous, infectious particles devoid of genetic material, are transmissible pathogens that cause neurodegenerative disorders in humans and animals [1]. Prions show strikingly different biochemical and biophysical properties from other pathogens, such as fungi, bacteria, and viruses, as well as differing host-pathogen interactions. Prions are unusually resistant to many conventional chemical and physical treatments to reduce infectivity, such as intensive ultraviolet radiation, heat, and nuclease treatment [2]. Prion infection induces no humoral or innate immune responses in the host [3]. Prions are also peculiar in their multiplication, which is not based on the central dogma of molecular biology but involves protein-protein interactions followed by conformational conversion. This represents a novel paradigm for propagation of infectious agents [4, 5].

Prions are composed of pathogenic scrapie prion protein (PrP^Sc^), a misfolded isoform of cellular prion protein (PrP^C^) [6]. PrP^C^, which is encoded by the* PRNP* locus, is comprised of 253 amino acids and tethered on the plasma membrane through a glycosylphosphatidylinositol anchor, with a di-, mono-, or unglycosylated state [7]. PrP^C^ is most abundant in neuronal cells [8], but it is also expressed in nonneuronal brain cells, such as astrocytes, microglia, and oligodendrocytes [9]. Although the level is relatively low, the expression of PrP^C^ is ubiquitous in noncentral nervous system cells, including some lymphocytes, hematopoietic progenitor cells, neuronal cell bodies of the olfactory epithelium, neuroepithelial bodies of the lung parenchyma, epithelial cells of the renal medulla, Sertoli cells, spermatocytes, hair follicle cells, and myocytes [10]. The physiological role of PrP^C^ has not been confirmed, although it has been studied using PrP^C^-deficient mice. However, a growing number of reports have proposed a putative function of PrP^C^ in development and maintenance of neurons, cell adhesion, and the maintenance of copper homeostasis [11–13]. Interestingly, PrP^C^ was shown to be involved in toxic signaling for pathogenic events linked with Alzheimer's and Parkinson's diseases [14–16].

Although the covalent biochemical properties of PrP^C^ and PrP^Sc^, including the primary structure and glycosylation state, are identical, their secondary and tertiary structures become distinct once PrP^C^ undergoes misfolding and is converted into PrP^Sc^. PrP^C^ contains ~40%  *α*-helical and ~3%  *β*-sheet motifs, whereas PrP^Sc^ is composed of ~30%  *α*-helical and ~40%  *β*-sheet motifs [17]. During the conformational transition, PrP^Sc^ serves as a template for alteration of the conformation of PrP^C^ to that of PrP^Sc^. This conversion changes a number of the properties of PrP molecules, such as solubility/hydrophobicity, protease sensitivity, antibody reactivity, and infectivity [18–20]. The increase in the number of PrP^Sc^ conformers containing a high content of *β*-sheet motifs gives rise to hydrophobic PrP^Sc^ aggregates.

## 2. Prion Disease

Prion diseases, also known as transmissible spongiform encephalopathies, comprise a group of incurable fatal neurologic disorders caused by prions. Creutzfeldt–Jakob disease (CJD) in humans, bovine spongiform encephalopathy (BSE, publicly known as “mad cow disease”) in cattle, scrapie in sheep, and chronic wasting disease (CWD) in elk and deer belong to prion diseases [22]. Although their origin and infection routes remain obscure, human prion diseases are considered to be caused by three different reasons. First, sporadic prion diseases, including sporadic CJD (sCJD), arise from spontaneous occurrence of PrP^Sc^ in the host [22, 23]. Second, genetic prion diseases, including familial CJD (fCJD), fatal familial insomnia (FFI), and Gerstmann–Sträussler–Scheinker syndrome (GSS), are caused by mutations of the* PRNP* gene, which increase the tendency for mutated PrP^C^ to be misfolded into the abnormal PrP^Sc^ [24, 25]. Finally, acquired prion diseases are caused by ingestion of prion-tainted material (variant CJD [vCJD]) or exposure to external prions via blood transfusion, tissue transplantation, hormone therapy, or surgical equipment (iatrogenic CJD [iCJD]) [26, 27].

Prion diseases are transmitted within various mammalian host species but can also spread between species, as demonstrated by transmission of BSE prions in humans [27]. The possibility of foodborne and iatrogenic prion transmission, as well as the steady prevalence of sCJD, is a potential concern for human public health, while regional expansion of CWD and occurrence of atypical animal prion diseases pose serious problems for animal health [28–31].

Prion disease has a long incubation period, from months to decades, and finally manifests as rapidly progressing dementia with severe neurodegeneration, ataxia, and involuntary movements at the clinical phase [32]. Additionally, affected hosts show neuropathological characteristics including spongiform vacuolation, neuronal loss, astrogliosis, and deposition of PrP^Sc^ in the brain tissue [33]. Occasionally, PrP^Sc^ deposits are found as large rod-like amyloid plaques [34]. While PrP^Sc^ is detected at the highest level in the central nervous system, abnormally folded PrP can also be found in the lymphoreticular and muscular systems [35]. It is also important to note that PrP^Sc^ can be detected in bodily fluids or excretions, such as saliva, feces, urine, and blood [36–38].

## 3. Approaches for Diagnosis of Prion Disease

Diagnosis of human prion disease is based on multiple criteria (reviewed in [39]). Clinical and neurological surveys are key to diagnosing prion disease. In clinical circumstances, patients have been diagnosed with prion disease following clinical presentation of several neurologic signs [40]. In addition, abnormalities in electroencephalography (EEG), magnetic resonance imaging (MRI), and cerebrospinal fluid (CSF) protein analyses are often used in clinical settings [41–43]. EEG detects abnormal patterns such as periodic sharp and slow wave complexes in sCJD patients [44]. MRI detects characteristic signal changes in specific areas of the patient's cerebral cortex with a relatively high sensitivity and specificity [43].

PrP gene analysis for known familial* PRNP* mutations is performed to diagnose genetic prion diseases [25, 45, 46]. Similar analysis is also useful for vCJD diagnosis, which can detect codon 129 (M/V) polymorphisms [47].

Neuropathologically, diagnosis of prion disease depends on detection of pathologic changes in the brain tissue, such as spongiosis, neuronal cell loss, astrocytic gliosis, and PrP^Sc^ deposition, using histological and immunohistochemical methods [40].

In biochemical laboratory settings, PrP^Sc^, the most reliable marker of prion disease, can be identified on the basis of its protease-resistant properties [2]. Western blotting and ELISA are the most widely used platforms to detect protease-resistant PrP^Sc^. Variant approaches include the cell lifting assay, scrapie cell assay, and histoblotting [48]. Because anti-PrP antibodies are key for biochemical analysis of PrP^Sc^, antibody sensitivity is critical for improving the sensitivity of detection [3]. Moreover, biochemical detection of elevated levels of 14-3-3, tau, neuron-specific enolase, and S100B in CSF of patients can be utilized for diagnostic purposes [42, 49].

All these approaches, however, are useful only postmortem or during the terminal stage of disease at the earliest. No method is currently available for diagnosis of prion disease at the preclinical stage. Because prions can evade the host immune system, many diagnostic tools, such as nucleic acid detection of pathogens by PCR amplification and detection of immune response changes by ELISA, are inadequate for biochemical detection and diagnosis of prions. Due to the potential risk of prions to public health and, more specifically, the poorly understood nature of prion pathogens, preemptive measures are necessary to prevent future outbreaks of prion diseases on a massive scale. Therefore, development of an ultrasensitive prion detection technique is critical for early diagnosis of prion diseases.

The most desirable tool for prion diagnosis should be the fully practical and sensitive assays for routine detection of prions, so that early diagnosis becomes feasible. Recently, marked progress has been made in the diagnostic process using novel ultrasensitive seeding assays. The seeding activity of PrP^Sc^ can be determined using a number of similar assays (Table 1). The amyloid seeding assay (ASA) and the PrP aggregate formation assay (PAFA) measure the seeding activity of PrP^Sc^ to generate PrP amyloids and aggregates in the presence of partially molten recombinant PrP (rPrP) [50, 51]. The protein misfolding cyclic amplification (PMCA) assay multiplies PrP^Sc^ seeds by converting PrP^C^ under undefined conditions [52]. The quaking-induced conversion (QuIC) assay is similar to the ASA and PAFA in measuring the seeding activity of PrP^Sc^ but differs in using an ionic detergent, such as sodium dodecyl sulfate (SDS), instead of a denaturant [53]. Application of thioflavin T (ThT), a fluorescent dye that quantifies the presence of fibrillated aggregates or amyloids of misfolded protein, to the ASA, PAFA, or QuIC permits real-time measurement of the seeding activity of PrP^Sc^ [21]. Currently, the real-time QuIC (RT-QuIC) assay is being intensively studied to investigate whether it is suitable to detect prion infection with high sensitivity and specificity.

## 4. RT-QuIC

The RT-QuIC assay shows promise for prion diagnosis, with a diagnostic sensitivity of 96% and a specificity approaching 100%, tested in detecting PrP^Sc^ in CSF samples of CJD patients [54]. In addition, RT-QuIC detected all subtypes of CJD in brain samples using minute amounts of seeds, up to femtogram units through end-point dilution [21, 46].

### 4.1. Basic Concept of RT-QuIC

The RT-QuIC assay to detect amyloid fibrils formed from misfolded rPrP substrates is an ultrasensitive detection technique developed from seeding assays based on prion-seeded conversion of *α*-rPrP refolded to resemble PrP^C^ [53, 55]. This assay involves cycles of vigorous shaking and incubation in 96-well plates. Soluble rPrP expressed in* E. coli* is used as a substrate to amplify the seeded PrP^Sc^, resulting in formation of aggregates and amyloid fibrils [56]. During incubation, PrP^Sc^ acts as a seed, inducing conformational change of *α*-rPrP substrates into amyloids and integrating them into a growing amyloid fibril. These rPrP aggregates can be detected with fluorescence plate readers, using the amyloid-sensitive dye ThT. Detection of the seeding assay products through ThT fluorescence reading in real time serves as the basis of RT-QuIC. Shaking, a key factor in this assay, promotes fragmentation of the amyloid fibrils, forming more reactive seeds. These seeds recruit more rPrP substrates, inducing more conversion and thus resulting in an exponential increase in amyloid formation.

### 4.2. Factors Affecting RT-QuIC Kinetics

Since the assay was developed, it has been optimized to reduce the formation of spontaneous ThT-positive amyloid fibrils and to enhance the detection limit [21, 57, 58]. Further optimization and standardization of the RT-QuIC assay are required for it to become a sufficiently specific and sensitive assay for being adopted as a routine diagnostic test because several factors affect the reactions of RT-QuIC [59].

Orrú et al. investigated a range of factors that may affect RT-QuIC [54]. The parameters include truncation of rPrP substrates, temperature, shaking speed, shaking interval, pH, and concentrations of denaturant and detergent. This study reported that use of N-terminal truncated hamster rPrP (residues 90–231) substrate produced a shorter lag phase than the full-length hamster rPrP (residues 23–231), which is consistent with their previous report. While the mechanism is not clear, the authors speculate that the absence of the flexible N-terminal residues 23–89 destabilizes the native rPrP conformation, thus allowing it to more rapidly refold into the amyloid conformation.

Since RT-QuIC is carried out in a controlled temperature environment using shaking-incubation cycles, temperature and shaking speed, as well as shaking interval, affect the assay [45]. Increased temperature promotes a faster RT-QuIC response. When the temperature was increased from 42°C to 60°C, the time to reach a maximum ThT fluorescence reading was reduced by 2- to 3-fold. An increase in shaking speed from 700 rpm to 1100 rpm not only shortened the lag phase but also allowed more consistent signal detection at the highest seed dilution. Shaking interval is a crucial factor in producing more reactive seeds via fragmentation of rPrP^Sc^ polymers and in enhancing the interaction between rPrP and PrP^Sc^ by promoting partial unfolding of rPrP [60, 61]. Although longer shaking promotes faster formation of amyloids, continuous shaking without a rest period is not recommended, as it induces spontaneous reactions that lead to false-positive signals. Increasing the shake-rest ratio while maintaining a short rest period within a two-minute cycle accelerated the RT-QuIC response; 100 s of shaking followed by 20 s of incubation produced the shortest lag phase.

The concentrations of denaturant and detergent affect the effectiveness of RT-QuIC. Guanidine hydrochloride (GdnHCl) was first thought to be required for the conversion of PrP^C^ to PrP^Sc^ in a cell-free system [62]. However, conversion of rPrP^C^ to rPrP^Sc^ still occurred in the absence of GdnHCl, and unseeded reactions exhibit a marked delay in spontaneous rPrP^Sc^ formation [21]. Therefore, the use of GdnHCl-free buffer is recommended as it enhances the sensitivity of RT-QuIC and reduces the risk of false-positive responses. Atarashi et al. reported that fibrils formed in the presence of SDS were much larger and thicker than those produced in the absence of SDS and that SDS enhanced the false-positive response [56]. The effect of SDS on false-positive responses was associated with rPrP substrates in the RT-QuIC reactions [54, 63]. Reduction of the SDS concentration significantly decreased the false-positive response when tested with mouse rPrP (23–231). Thus, a low concentration of SDS is preferable for optimal detection of mouse-adapted scrapie prions and to decrease the rate of false-positive responses.

The presence of salt is crucial for rPrP^Sc^ formation in cell-free conversion in the absence of GdnHCl. The sensitivity of RT-QuIC was maximal at 500 mM NaCl at pH 7.4 [58]. The pH condition also influences RT-QuIC. Lowering the pH of the assay elongated the lag phase, compared to performance at the standard pH 7.4. In this context, Atarashi et al. recently modified the RT-QuIC, using a buffer supplemented with a chelating agent and a high salt concentration at neutral pH, without SDS [21].

### 4.3. Presentation and Analysis of RT-QuIC Data

**Figure 1:**
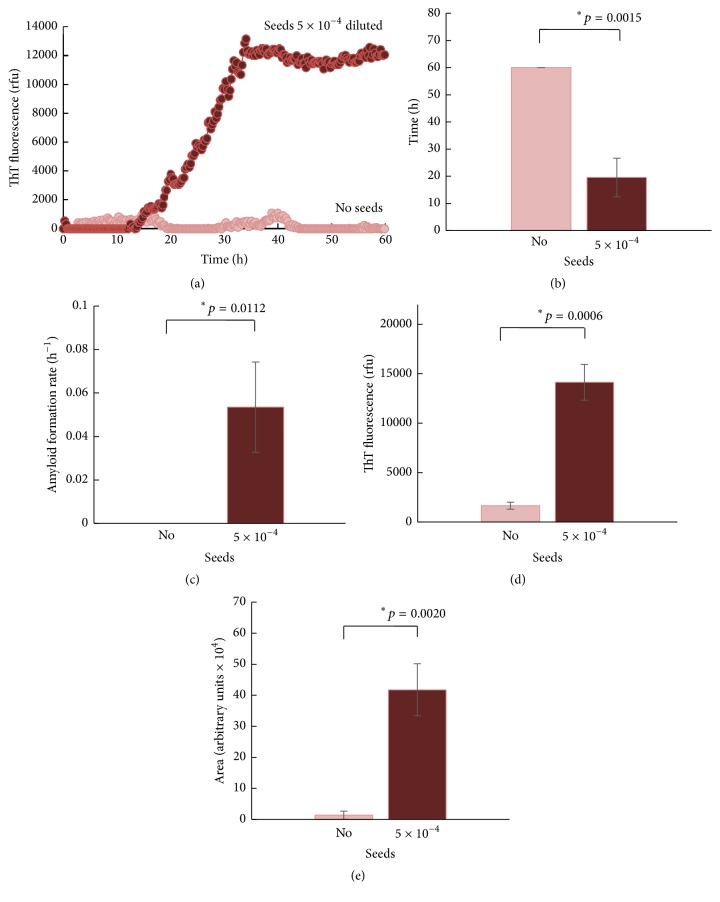
RT-QuIC data. (a) A representative RT-QuIC response. A set of RT-QuIC quadruple reactions was performed with mouse rPrP (89–231) and PrP^Sc^ seeds from ScN2a cells diluted 5 × 10^−4^-fold. RT-QuIC was performed using a modified version of the method described by Atarashi et al. [21]. The average ThT fluorescence was plotted against assay time. (b) Differences in length of lag phase. (c) Differences in amyloid formation rate. (d) Differences in ThT fluorescence maxima. (e) Differences in integrated area under the curve. Averages and standard deviations (error bars) were calculated using multiple data sets (*n* = 4). Student's *t*-test was used for statistical analysis. A *p* value < 0.05 (*∗*) was considered statistically significant.

The relative seeding activities of the test sample can be presented by graphing fluorescence readouts against assay time (Figure 1(a)). Quantitative measures that can be used to analyze the RT-QuIC data include lag phase length, amyloid formation rate, maximal signal intensity, and area under the RT-QuIC curve (Figures 1(b)–1(e)). Because ThT fluorescence was measured in real time every few minutes, the time at which the readouts are above the average fluorescence values of the control reactions can be determined and compared for the length of lag phase [21]. Similarly, the cut-off time (Ct) values can be calculated by determining the time when each positive reaction exceeded a threshold (5 standard deviations above the mean initial fluorescence). The amyloid formation rate is expressed as the inverse (1/time to threshold) of the Ct [64]. The ThT fluorescence maxima in the stationary phase of real-time readouts can be a simple, reliable indicator to reflect seeding activity measured in the RT-QuIC [21]. The area under the RT-QuIC curve, calculated by integration, is used to express RT-QuIC efficiency because it reflects a combined measurement of rapidity of seeding activity and quantity of aggregates formed [65]. Different methods for expression of seeding activity in RT-QuIC (Figure 1) unambiguously distinguish the presence or absence of PrP^Sc^ seeds.

### 4.4. Application of RT-QuIC

Since the development of RT-QuIC, its application has been expanded to several areas of prion research including diagnosis of human and animal prion diseases, prion strain differentiation, and a titration assay for prion infectivity, as an alternative to animal bioassay.

#### 4.4.1. RT-QuIC for Human Prion Diseases

RT-QuIC was first established as a diagnostic tool for human prion disease using CSF samples. Initially, the RT-QuIC showed greater than 80% sensitivity and 100% specificity in a study of Japanese and Australian subjects with CJD or without CJD but with other neurological diseases [21]. The results were confirmed in independent investigations, in which this assay was regarded as sufficient as an antemortem diagnostic test using CSF specimens, showing 96% sensitivity and 100% specificity for diagnosing sporadic CJD [54, 66, 67]. To assess the reproducibility of the RT-QuIC in diagnosing human prion diseases, international ring trials were conducted [49, 68]. A total of 86 CSF samples, including 32 non-prion disease controls, were examined in two laboratories, and 6 samples, including 5 controls, were investigated in four laboratories. These two tests showed almost perfect agreement between the different participating sites [68]. Another international ring trial was undertaken by another group of researchers, in which a set of 25 CSF samples were analyzed by a total of 11 different centers in 8 countries. This investigation showed almost complete concordance between the centers, suggesting that RT-QuIC is a suitably reliable and robust technique for clinical practice [49]. Sano and colleagues evaluated the RT-QuIC assay using 56 CSF samples from patients with genetic prion disease, including 20 cases of GSS with a P102L mutation, 12 cases of FFI with a D178N mutation, and 24 cases of fCJD, comprising 22 cases with an E200K mutation and 2 with a V203I mutation [45]. The RT-QuIC assay was more sensitive than the biomarker test for 14-3-3 in diagnosing genetic prion disease patients with detection sensitivities of 78% for GSS, 100% for FFI, 87% for fCJD E200K, and 100% for fCJD V203I, suggesting that this assay is a useful diagnostic tool for detection of genetic human prion diseases.

Subsequently, the RT-QuIC was shown to detect PrP^Sc^ in the lining of the nasal surface, which led to the testing of olfactory nasal brushing samples. Olfactory neural cells are the only surface neural cells of the body, and the olfactory mucosa could be considered as a window to the brain [69]. Compared to CSF samples, nasal samples can be obtained using a less invasive method, with reduced risk of contamination of samples with blood, which inhibits the reaction. RT-QuIC revealed high seeding activity of sCJD in the olfactory mucosa. The RT-QuIC response using olfactory nasal brushing samples was faster and stronger than using CSF samples. This approach, providing 97% sensitivity and 100% specificity, supports establishment of a method for relatively simple antemortem diagnosis of CJD [70].

#### 4.4.2. RT-QuIC for Animal Prion Diseases

In addition to human prion disease, the RT-QuIC is also a highly useful method to detect animal prion diseases, especially CWD [37, 38, 71–75]. CWD is the only prion disease affecting free-ranging animals. Unlike in most other prion diseases, CWD prions are shed in bodily fluids, resulting in horizontal transmission within and between cervid species [31]. The affected geographical area is expanding as recently shown by the first CWD outbreak in Europe [30]. The RT-QuIC assay was applied for diagnosis of CWD using various types of specimens: CSF, nasal brushing, lymph node, and rectoanal mucosa associated lymphoid tissue (RAMALT) [71–73, 76]. The RT-QuIC detected CWD prions in RAMALT biopsy specimens with a sensitivity of 77.3%, although the assay results depended on the species, disease progression, genotype of* PRNP*, and specimen. More interestingly, different types of samples, such as blood, saliva, urine, and feces, were used to detect prion disease using the RT-QuIC assay [37, 38, 77, 78]. CWD prions were detected in urine and fecal extracts collected from presymptomatic deer [37]. Furthermore, CWD prions were detected in 14 of 24 (58.3%) diluted saliva samples from CWD-exposed white-tailed deer, including 9 of 14 asymptomatic animals (64.2%). In addition, phosphotungstic acid enrichment enhanced the sensitivity of the RT-QuIC assay, enabling detection in 19 of 24 (79.1%) saliva samples used above [38]. Interestingly, the RT-QuIC assay can amplify, detect, and quantify the amyloid seeding activity of CWD prions in fixed paraffin embedded (FPE) tissue sections with greater detection sensitivity than immunohistochemical analysis of tissues or semiquantitation of prions in a given FPE tissue [78]. Taken together, RT-QuIC is useful for both diagnosis and surveillance of CWD.

#### 4.4.3. Use of RT-QuIC for Prion Strain Typing and Infectivity Titration

The RT-QuIC can also be used in prion strain typing, as different strains show slight differences in RT-QuIC response [63, 65, 74]. Cramm and colleagues demonstrated that the seeding activity of PrP^Sc^ differs between various human prion diseases [65]. These differences in RT-QuIC allow the identification of unknown strains in patients. In a recent report, full-length bank vole rPrP was suggested as an apparently universal substrate for RT-QuIC-based detection. Using this approach, prion strains could be discriminated: for instance, classical and atypical L-type BSE; classical and atypical Nor98 sheep scrapie; and human sCJD and vCJD. [63, 75]. In fact, L-BSE was detected using multiple rPrP substrates, while C-BSE was much more selective [74].

The RT-QuIC has also been developed to allow prion infectivity titration, which has classically been performed by bioassays. The sensitivity of RT-QuIC-based titration is similar to that of bioassays, while the assay requires greatly reduced time and cost [57, 64]. RT-QuIC analysis of CWD prions showed that serial dilution of prion seeds was linearly related to the rate of amyloid formation over a range of 10^−3^ to 10^−8 ^*μ*g. Owing to this linear correlation, a standard curve for the amyloid formation rate of reference samples (CWD-positive brain homogenate previously titrated by bioassay) can be established to estimate the prion seed concentration and infectivity in tissues, bodily fluids, and excreta [64].

## 5. Conclusion

PrP^Sc^ multiplies by protein interaction with PrP^C^ and subsequent conformational conversion. PrP^Sc^ then forms aggregates, such as fibrils and amyloids. The RT-QuIC mimics these biochemical events. The RT-QuIC has demonstrated high sensitivity and specificity for detecting seeding activity of PrP^Sc^ in both human and animal samples. It is expected that incorporation of RT-QuIC as an additional criterion for diagnosis and surveillance of prion diseases, together with the current standard, would advance the status of prion diagnosis. The RT-QuIC requires further improvement for a few issues, such as reduction of the invasiveness of sample collection, expansion of adequate samples used for assays, ease of sampling, maintenance of the least false-negative responses, abundant availability of appropriate rPrP, and standardization of criteria defining RT-QuIC responses and the experimental environment. Nevertheless, this assay represents a powerful addition to the efforts to establish a prion diagnostic tool. In addition, the principle of RT-QuIC can easily be applied to diagnosis of other neurological diseases associated with protein misfolding and aggregation.

## Figures and Tables

**Table 1:** Assays to measure PrP aggregation and seeding activity of PrP^Sc^.

	Substrate	Denaturant	Detergent	Product	Detection method	Real-time detection
ASA/PAFA	Recombinant PrP (*α*-PrP^§^)	Guanidine-HCl	No	Aggregates, amyloid	ThT fluorescence	Yes
PMCA	PrP^C^ from cells or tissues	No	Triton X-100	PrP^Sc^	WB for PK-resistant PrP^Sc^	No
RT-QuIC	Recombinant PrP (*α*-PrP^§^)	No	SDS	Aggregates, amyloid	ThT fluorescence	Yes

^§^ *α*-PrP prepared from recombinant PrP with a high *α*-helical content. SDS: sodium dodecyl sulfate; WB: western blotting; PK: proteinase K; ThT: thioflavin T.
